# Transfer of Low-Density Lipoproteins in Coronary Artery Bifurcation Lesions with Stenosed Side Branch: Numerical Study

**DOI:** 10.1155/2019/5297284

**Published:** 2019-10-15

**Authors:** Zhenmin Fan, Xiao Liu, Peng Zhang, Jiang Gu, Xia Ye, Xiaoyan Deng

**Affiliations:** ^1^School of Mechanical Engineering, Jiangsu University of Technology, Changzhou, Jiangsu 213001, China; ^2^Key Laboratory for Biomechanics and Mechanobiology of Ministry of Education, School of Biological Science and Medical Engineering, Beihang University, Beijing 100191, China

## Abstract

Evidence from clinical data suggests that the stenotic side branch (SB) is one of the key predictors for SB occlusion-based adverse events. In this study, we hypothesized that coronary bifurcations with stenotic SB might lead to severe concentration polarization of atherogenic lipids, such as the low-density lipoproteins (LDL), motivating the adverse events in the clinic. To confirm this hypothesis, this work numerically investigated the transport of LDL in different bifurcation lesions based on the Medina classification with various location and stenosis severities. The results showed that the coronary bifurcations with stenotic SB might be suffering more serious concentration polarization of LDL on the luminal surface of the SB due to higher level of LDL concentrations. Moreover, compared to the other bifurcation lesion types, the type (1,0,1) had the highest luminal surface LDL concentration along the SB and the highest degree of risk to enhance the process of atherosclerosis. In addition, this study also showed that the luminal surface LDL concentration increased with elevated stenosis severity. The type (1,0,1) with the severe stenosis (75% diameter reduction) had the highest concentration at the SB. In conclusion, these results suggested that both location of lesions and stenosis severities had great influence on the distribution of LDL on the luminal surface of the SB. Therefore, the estimation of disease severity and the interventional therapy should be carried out not only according to the stenosis severities in clinic. Moreover, compared to the other bifurcation lesion types, the type (1,0,1), rather than the type (1,1,1) as usually considered, had the highest luminal surface LDL concentration along the SB and the highest degree of risk to enhance the process of atherosclerosis.

## 1. Background

Coronary bifurcation lesions are associated with adverse clinical outcomes (e.g., myocardial infarction). Numerous clinical data have provided evidence that the stenotic side branch (SB) is one of the key predictors of clinical adverse events [1, 2, 3]. Furthermore, it has been shown that disturbed blood flow in or near side branches is associated with the occurrence of vascular inflammation and atherosclerosis [4, 5]. Early studies have also demonstrated that disturbed or oscillatory flows near an arterial bifurcation play a critical role in stenotic SB occlusion [4, 5, 6]. Nevertheless, due to the accumulating atherogenic lipids on the arterial wall leading to the genesis of atherosclerosis, researchers typically focus on the mass transfer, such as low-density lipoproteins (LDL) [7]. To study the phenomenon of atherogenesis localization in the circulatory system, Liu et al. theoretically and experimentally showed that the concentration polarization of lipid was present in the arterial system, which had a great relationship with the formation of atherosclerosis [8, 9].

**Figure 1:**
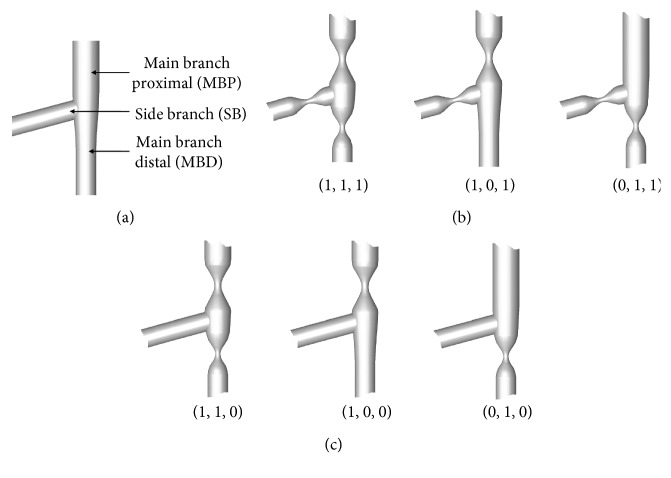
(a) Idealized geometric models of coronary artery bifurcations. (b) Coronary bifurcation models with stenotic SB and (c) models without with stenotic SB.

Coronary bifurcation (Figure 1(a)) is a typical blood vessel of complex geometry characterized by branching. It generally consists of the SB, the main branch distal (MBD), and the main branch proximal (MBP). The bifurcation lesions of coronary are defined as any branch with a reduction that exists among the branches. Bifurcation lesions have been commonly categorized based on the Medina classification system according to the location of the luminal narrowing [10]. In this classification system, any branch with a reduction is marked by the binary value 1, or else it is denoted by the value 0. In clinic, this classification has been widely approved to describe coronary bifurcation lesions.

Regarding the difficulties in the analysis of blood flow in patients, numerical simulations on coronary bifurcation were performed to better understand its hemodynamic changes [6, 11]. The Medina classification and some studies indicated that the type (1,1,1) of bifurcation lesion would be the greatest risk type of branch. However, few studies have explored the coronary bifurcation from the point of view in mass transfer or spatial distribution of some lipids, particularly atherogenic lipids, such as LDL. This study supposed that the abnormal distribution of luminal surface LDL concentrations presented in the stenotic SB might have great effect on the degree of atherogenesis and therefore on the SB occlusion. To confirm this hypothesis in this work, models of coronary bifurcation with various locations of lesions were established according to the Medina classification. Additionally, the mass transport of LDL in the SB was numerically investigated to evaluate the cause of the SB occlusion. The effects of the multilesion configurations and stenosis severity on the critical SB luminal surface LDL concentrations were also discussed.

## 2. Methods

### 2.1. Geometry of Stenotic Bifurcations

**Figure 2:**
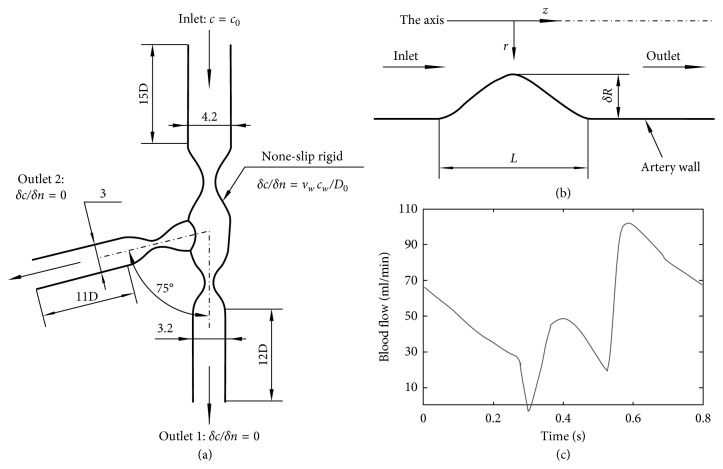
(a) Schematic of the computational geometry with boundary conditions. (b) Schematic of the computational stenosis. (c) Inlet fluid velocity waveform in this study [12].

The coronary bifurcation configurations (Figure 1) from Frattolin et al. [6], presented in this study, were made up of the left anterior descending artery, the left main coronary artery, and the left circumflex artery bifurcation. The idealized coronary arteries (Figure 2(a)) in all the models were 3 mm for the SB, 4.2 mm for the MBP, and 3.2 mm for the MBD [11]. To satisfy Finet's law of coronary bifurcations, idealized coronaries were set with a fractal ratio (the mother-vessel diameter to the sum of the two daughter-vessel diameters) of 0.67 [13]. The bifurcation angle, which is the deviation angle between the SB and the MBD, was 75°, in accordance with the dimensions of typical anatomical data [6]. Figures 1(b)-1(c) display the coronary bifurcation models that were utilized in this study.

As shown in Figure 2(b), the geometry of the luminal narrowing is axisymmetric with a stenosis in the transverse section of lumen. The form of the reduction in the arterial lumen is modeled by the following equation [14]:

(1) $$\begin{array}{c} \frac{r\left(Z\right)}{R}=1-\frac{\delta }{2}\left[1+\cos\left(\frac{2\pi Z}{L}\right)\right], \end{array}$$

where *r*(*Z*) is the radius of the arterial lumen at *Z*, while the throat of the narrowing is located at *Z* = 0. The length of the narrowing is *L*, and the diameter of the healthy region of the lumen is *D* and is chosen as the dimensionless radius reduction at the throat of the narrowing. The radius at the throat of the stenosis after dimensionless is *δ*. To investigate the influence of the stenosis severity and construct models associated with the Medina classification, *δ* is assigned 0.75, 0.50, and 0.25 to represent lesions of 25%, 50%, and 75% diameter stenosis, respectively.

For each model, the dimensionless radius at the stenosis was the same in the MBP, MBD, and SB. To achieve fully developed flow, the coronary bifurcation was prolonged 11D and 12D downstream and 15D upstream, respectively [8].

### 2.2. Assumptions

In this work, we assumed blood would be a homogeneous fluid that is an incompressible non-Newtonian liquid [15]. The vessel wall is nonslip rigid [16].

### 2.3. Governing Equations

Flow of numerical simulations is performed based on continuity equations and the incompressible Navier–Stokes [15, 16]:

(2) $$\begin{array}{c} \rho \left(\frac{\partial u}{\partial t}+u\cdot \nabla u\right)=-\nabla p+\nabla \cdot \tau ,\\ \nabla \cdot u=0, \end{array}$$

where *p* and *u* are the pressure and the fluid velocity vector, respectively. The density of blood is *ρ* = 1050 kg/m^3^, and the tension tensor is *τ*, which is described by

(3) $$\begin{array}{c} \tau =2\eta \left(\dot{\gamma }\right)T, \end{array}$$

where $\dot{\gamma }$ and *T* are the shear rate and deformation tensor, respectively, and the viscosity of blood is *η*, which is regulated by the shear rate. The Carreau model is applied to obtain the viscosity of blood.

(4) $$\begin{array}{c} \eta \left(\overset{˙}{\gamma }\right)=\eta_{\infty }+\left(\eta_{0}-\eta_{\infty }\right){\left[1+{\left(\lambda \overset{˙}{\gamma }\right)}^{2}\right]}^{\left(\left(n-1\right)/2\right)} \end{array}$$

where *η*_0_ = 5.6 × 10^−2^ kg/(m s), *η*_*∞*_ = 3.45 × 10^−3^ kg/(m s), *λ* = 3.313 s, and *n* = 0.3568 [17]. The mass transfer of LDL is described by the following convection-diffusion equation:

(5) $$\begin{array}{c} \frac{\partial c}{\partial t}+u\cdot \nabla c-D\Delta c=0, \end{array}$$

where *D* = 4.8 × 10^−12^ m^2^/s represents the diffusion coefficient of LDL, and the concentration of LDL is *c* [18].

### 2.4. Boundary Conditions

Both the flow and LDL transport boundary conditions are depicted in Figure 2(b).

The boundary conditions for the flow simulation are as follows [8, 12, 19]:

  Inlet: a mean velocity waveform and the parabolic flow velocity profile which is time dependent shown in Figure 2(c) is applied at the inlet
  Outlet: outflow boundary condition with a zero gauge pressure is set at both the outlets [20]
  Wall: the arterial wall is assumed as the nonslip-rigid wall

The mass transport of LDL is described by [8]

(6) $$\begin{array}{c} \text{Inlet}:c=c_{0}=2.86\times 10^{-2}\text{mol}/{\text{m}}^{3};\\ \text{Outlet}:\frac{\partial c}{\partial n}=0;\\ \text{Walls}:v_{\text{w}}c_{\text{w}}-D\left(\frac{\partial c}{\partial n}\right)=0; \end{array}$$

where the respective LDL concentrations at the luminal surface of the arterial wall and the bulk flow are *c*_w_ and *c*_0_, respectively, and *n* represents the unit vector normal to arterial wall. The water filtration rate of artery is *v*_w_, which is set to 4.2 × 10^−8^ m/s [8].

For a better comparison and analysis of data, the presentation of the results is time averaged concentration of the LDL (*c*). That is defined as follows:

(7) $$\begin{array}{c} c=\frac{1}{T}\int_{0}^{T}c_{t}\text{ d}t, \end{array}$$

where *t* stands for the time, *T* is duration per pulse, and *c*_*t*_ is concentration of the LDL at the time of *t*.

### 2.5. Computation Procedures

These numerical calculations were performed by ANSYS Fluent. We employed a user-defined function to solve the Navier–Stokes and the mass transfer equation, which were validated by Liu et al. [8]. All the results in this study are somewhat mesh independent. At the beginning of the calculations, the time-averaged inlet velocity was applied as the initial boundary conditions for the pulsatile flow calculation. To get rid of the start-up effects on the transient fluid, the results are obtained at the fifth cycle. Additionally, the calculations are carried out with the convergence criterion 10^−5^ and time-step size 0.01 s.

## 3. Results

### 3.1. Effect of Lesion Location on Distribution of LDL (*c*_w_/*c*_0_) in the Coronary Bifurcation

**Figure 3:**
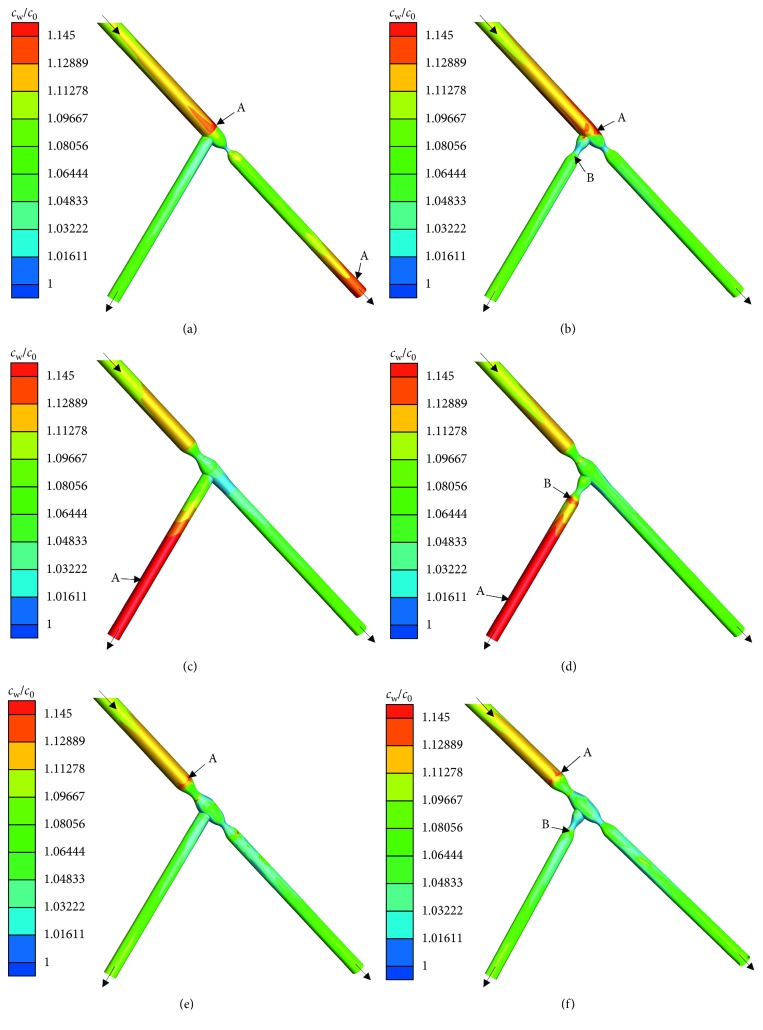
Distribution of luminal surface LDL in the coronary bifurcation lesions (lesion type (a) (0,1,0), (b) (0,1,1), (c) (1,0,0), (d) (1,0,1), (e) (1,1,0), and (f) (1,1,1)). Regions A and B have relatively serious concentration polarization of LDL.

The first set of simulations investigated the effect of the lesion location on the distribution and magnitude of the LDL. The coronary bifurcations with 75% diameter obstruction were analyzed according to the Medina classification, and the distributions of luminal surface LDL in the coronary bifurcations are shown in Figure 3. In general, the distributions of LDL on the luminal surface of coronary bifurcations were significantly different; however, the luminal surface of models was in higher level of LDL than the bulk flow (*c*_0_) in most regions of the bifurcations.

To determine the effects of stenosis in the SB, we firstly compared the lesion type (0,1,1) with the type (0,1,0) in Figures 3(a) and 3(b). The distribution of *c*_w_ was uneven in these bifurcations where *c*_w_ was in higher value along the MBP, especially the distal end of MBP (region A) where *c*_w_ were ∼16% higher than *c*_0_, while the distal end of MPD in the lesion type (0,1,0) was the highest in these bifurcations. However, in Figures 3(a) and 3(b), there was no much difference in the SB for these models in terms of the distributions of LDL where *c*_w_ was 5–9% higher than *c*_0_.

Secondly, we compared the lesion type (1,0,0) with the type (1,0,1) in Figures 3(c) and 3(d). The distribution of *c*_w_ was similar in these models, where *c*_w_ along the luminal surface of SB was much higher than other regions, especially the end of SB (region A) where *c*_w_ was 24% higher than *c*_0_ and was the highest in these bifurcations. However, the level of luminal surface LDL at the end of stenosis in the SB (region B) was much higher than the lesion type (1,0,0) at the same region without stenosis.

A comparison of the distributions of LDL on the luminal surface of the lesion type (1,1,0) and (1,1,1) in Figures 3(e) and 3(f) shows that the latter was in similar distribution of LDL as the former, indicating that the stenotic side branch generally had little effect on the distributions of LDL on the luminal surface of these models. *c*_w_ along the luminal surface of MBP were higher than other regions, especially the distal end of MBP (region A) where *c*_w_ were ∼15% higher than *c*_0_. However, the level of luminal surface LDL at the end of stenosis in the SB (region B) in the lesion type (1,1,1) was also much higher than the type (1,1,0) at the same region without stenosis.

Furthermore, in contrast to various models of the coronary bifurcations with stenosis in Figure 3, the lesion type (1,0,1) was in the most higher level of LDL on the surface of the SB.

**Figure 4:**
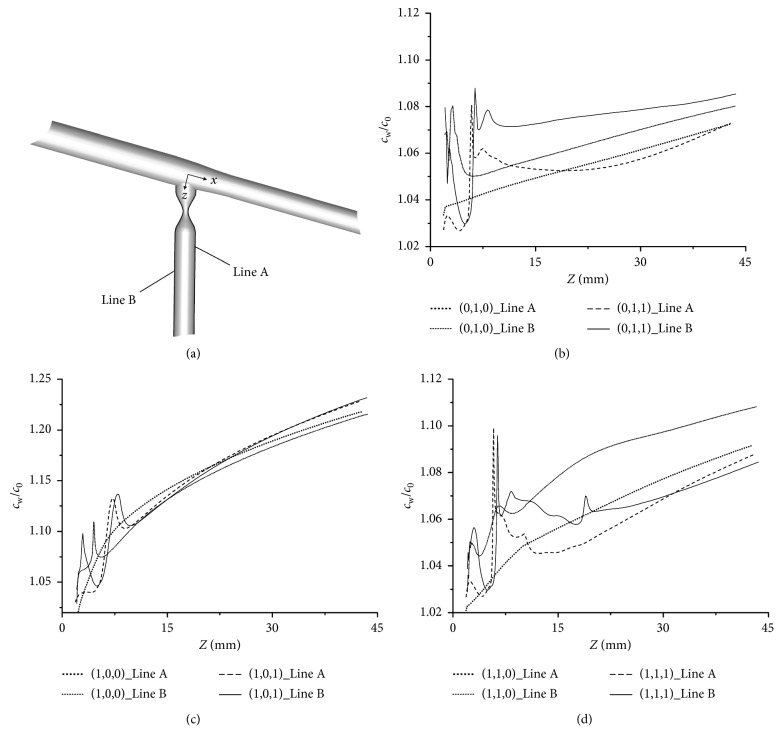
Effect of the location of lesions on the distribution of LDL along the SB. (a) Line A and Line B, which are located at the lumen surface, are along the inner wall and outer wall of the SB, respectively. (b) *c*_w_/*c*_0_ along lines in the type (0,1,0) and (0,1,1). (c) *c*_w_/*c*_0_ along lines in type (1,0,0) and (1,0,1). (d) *c*_w_/*c*_0_ along lines in type (1,1,0) and (1,1,1).

To facilitate the presentation of the LDL distribution in the stenotic models, the LDL were computed along the inner wall (Line A) and the outer wall (Line B) of the SB (Figure 4(a)).

Figure 4(b) shows that the acute fluctuation appeared along the Line A and Line B in the lesion type (0,1,0). At beginning of the SB, *c*_w_/*c*_0_ along Line A and Line B increased drastically, reaching a peak value of approximately 1.08 at the slightly lower reaches of the stenosis. After the peak value, *c*_w_/*c*_0_ decreased sharply, suffering another small fluctuation before the blood flowed out the stenosis. At the region of *Z* > 10 mm, *c*_w_/*c*_0_ increased slowly, reaching the second peak value at end of the SB. However, for the lesion type (0,1,1), there was an increase in *c*_w_/*c*_0_ along the Line B, after a fluctuation at the beginning of the SB (*Z* < 7.5 mm). The peak value of *c*_w_/*c*_0_ in lesion type (0,1,1) was approximately 1.09 at end of SB. A comparison between type (0,1,1) and type (0,1,0) in terms of the *c*_w_/*c*_0_ along the Line A and Line B demonstrated that the former generally had a higher *c*_w_/*c*_0_ than the latter. To be specific, the maximum difference was located at the stenosis in type (0,1,1) along the Line A with a 6.3% higher *c*_w_/*c*_0_ than the type (0,1,0). Furthermore, comparison of these models showed that *c*_w_/*c*_0_ along the outer wall was higher than that along the inner wall, and a peak difference was reached near the stenosis throat.

In Figure 4(c), for the lesion type (1,0,0), *c*_w_/*c*_0_ along Line A and Line B were similar as the type (1,0,1), which was with a stable increasing trend after a small fluctuation along the inner wall and outer wall of the SB. The maximum difference between these models was located at the stenosis in type (1,0,1) along the Line A with a 4.9% higher *c*_w_/*c*_0_ than type (1,0,0). *c*_w_/*c*_0_ along the Line B in the type (1,0,1) reached a peak value of approximately 1.23 at end of the SB.

Figure 4(d) shows that *c*_w_/*c*_0_ varied greatly along Line A and Line B in the lesion type (1,1,1). At the slightly lower reaches of the stenosis of the SB, *c*_w_/*c*_0_ increased drastically, reaching a peak value of approximately 1.98. After the peak value, *c*_w_/*c*_0_ decreased sharply, suffering another small fluctuation. At the region of *Z* > 20 mm, *c*_w_/*c*_0_ steady increased, reaching the second peak value at end of the SB. On the other hand, for the lesion type (1,1,0), there was an increase in *c*_w_/*c*_0_ both along the Line A and Line B, with a small fluctuation at the beginning of the SB (*Z* < 7.5 mm). The peak value of *c*_w_/*c*_0_ in lesion type (1,1,0) was approximately 1.11. A comparison between type (1,1,1) and type (1,1,0) in terms of the *c*_w_/*c*_0_ along the Line A and Line B demonstrated that the former generally was in higher *c*_w_/*c*_0_ than the latter. Similarly, comparison of these models showed that *c*_w_/*c*_0_ along the outer wall was higher than that along the inner wall, and a peak difference was reached near the stenosis throat.

As shown in Figures 4(b)–4(d), the profiles of *c*_w_/*c*_0_ in the stenotic SB were similar. Generally, the value of *c*_w_/*c*_0_ for the models with SB stenosis was higher than that for the models without stenosis, and the peak difference was near the stenosis throat. When compared with other types, the type (1,0,1) had the higher level of *c*_w_/*c*_0_ near the stenosis throat and most regions of the SB, and the second and third highest LDL concentrations were for type (0,1,1) and type (1,1,1), respectively. Furthermore, *c*_w_/*c*_0_ of all the models along the outer wall was higher than that along the inner wall.

**Figure 5:**
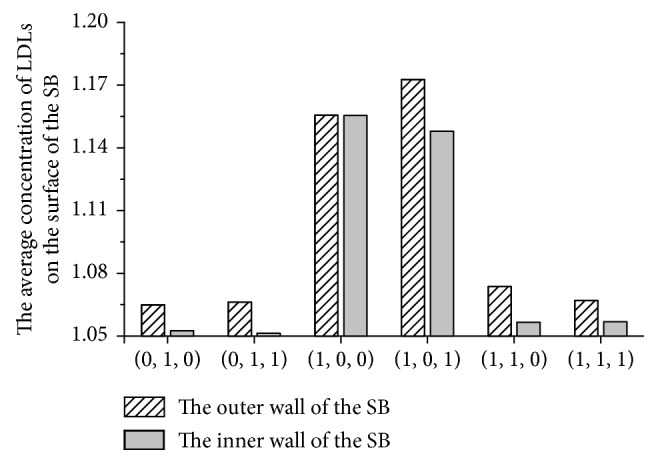
The average concentration of LDL on the surface of the SB.

To illustrate the variation in the LDL, the average *c*_w_/*c*_0_ along the lumen surface of the SB was computed (Figure 5). Generally, the average concentration on the stenotic SB is higher than that in the models without, and the peak value is for the type (1,0,1). The average *c*_w_/*c*_0_ on the SB of the type (1,0,1) and (1,0,0) were much higher than other types and were above 1.15. Furthermore, the average *c*_w_/*c*_0_ along the outer wall in all the models is higher than the inner wall as showed in the former results.

### 3.2. Effect of the Stenosis Severity on Distribution of *c*_w_/*c*_0_ in the SB

**Figure 6:**
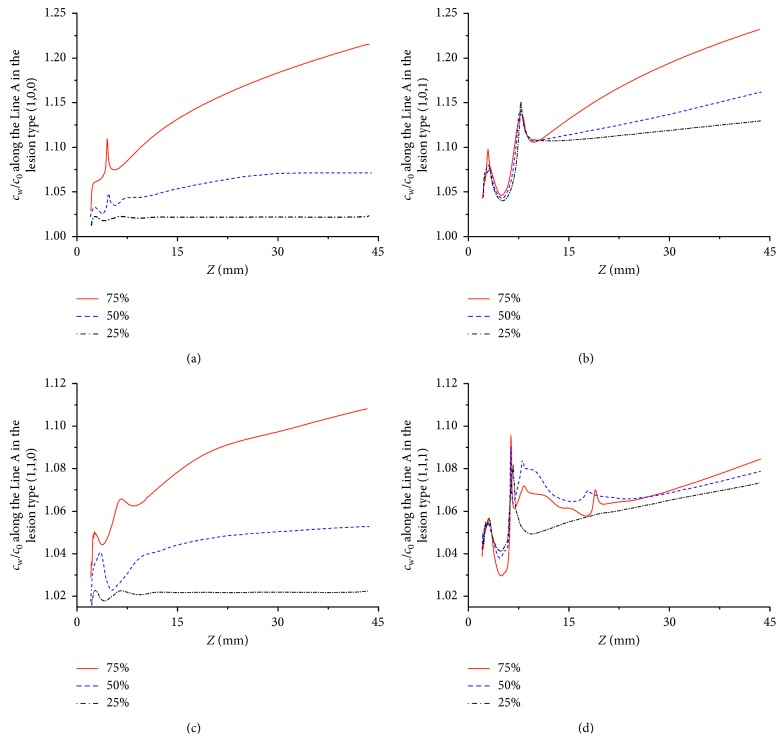
Effect of stenosis severity on the distribution LDL along the Line A in the lesion types (a) (1,0,0), (b) (1,0,1), (c) (1,1,0), and (d) (1,1,1).

The influence of stenosis severity was investigated using the percentage of the stenotic diameter that corresponded to mild and moderate severe lesions. Lesion types (1,0,0), (1,0,1), (1,1,0), and (1,1,1) with 25%, 50%, and 75% diameter stenosis were analyzed. The above results suggested that the outer wall of the SB was more vulnerable to the higher level of LDL. So, *c*_w_/*c*_0_ along Line A (at the outer wall of the SB) in various models were computed and are shown in Figure 6.

Generally, the trend of *c*_w_*/c*_0_ along Line A in all models was similar that *c*_w_/*c*_0_ table increased with *Z* after the small fluctuation at the beginning of the SB (*Z* < 7.5 mm), as shown in Figure 6. Comparison of the stenosis severity between different models suggested that the models with 75% diameter stenosis were had higher level of *c*_w_/*c*_0_ than those with 50% diameter stenosis, while the level of *c*_w_/*c*_0_ in the models with 50% was higher than those models with 25% diameter stenosis. These results indicated that the concentration of LDL increased with the growth of the stenosis severity, and lesion type (1,0,1) with 75% diameter stenosis was in the most higher level of *c*_w_/*c*_0_ on the whole.

For all the models, the magnitude and distribution of *c*_w_ along Line A changed greatly with the lesion severity. However, the lesion severity had relatively little impact on *c*_w_ at the region of stenosis in the lesion types (1,0,1) and (1,1,1), except the concomitant sharp increase of *c*_w_ at the beginning of the SB (*Z* < 7.5 mm). However, the magnitude of *c*_w_ along Line A in lesion types (1,0,1) and (1,1,1) significantly increased with stenosis degree of vascular lumen.

## 4. Discussion

Clinical data revealed that the SB occlusion is a factor in predicting adverse events. It is widely believed that abnormal hemodynamics, such as low shear stress, are associated with adverse events [4, 5]. Early investigations have focused on quantitatively investigating the effect of stenotic side branch on the distribution of wall shear stress (WSS) and the blood flow in coronary bifurcation lesions [4, 5]. However, these studies were carried out without considering the atherogenic lipids. Due to the fact that the deposition of atherogenic lipids within the artery is the first step to the genesis of atherosclerosis [7, 21], we hypothesized that the concentration polarization for lipids put forward by Deng et al. would be an explanation for SB occlusion. To confirm this hypothesis, the present study numerically investigated the transport of LDL in different bifurcation lesions based on the Medina classification.

Our simulation showed that the models with the stenotic SB generally had more serious LDL concentrations polarization along the stenotic SB than these models without the stenosis at the SB (Figures 4–6). This concentration polarization might lead to more severe accumulation or infiltration of LDL within the SB, resulting in an aggravation of the atherosclerosis at the SB. Hence, the high level of concentration polarization along the outer wall might be a more reasonable reason why SB occlusion is a predictor for adverse events in clinical data [3]. However, in comparison with the stenotic SB, the location of lesions has played a more important role in level of LDL in the SB. The numerical results found that lesion types (1,0,1), (1,0,0), and (1,1,1) had more serious LDL concentrations polarization along the stenotic SB than others (Figures 4–6). More concretely, the type (1,0,1) in bifurcation lesions was in the highest level of LDL concentration (Figures 4–6). There might be two reasons for these results. Firstly, the stenosis in the SB can significantly suppress the blood flow to the SB; in the meantime, the blood perfusion to the MBD elevated due to coronary branch steal. The second reason is that coronary with the stenotic SB has significant influence on the distribution of WSS in the SB. According to earlier numerical studies, coronary bifurcation lesions with the stenotic SB had the lower level of WSS in the SB [6, 22, 23]. Moreover, the present study showed that the type (1,0,1) might be most critical in clinic due to severe accumulation or infiltration of lipid within the SB, and clinical intervention should be carried out to prevent the further atherosclerotic disease. These results do not agree with the Medina classification, which indicated that the type (1,1,1) was the most severe and riskiest lesion. However, these results are consistent with the hemodynamics of earlier studies, which showed that both the reduction of the flow rate and WSS was greatest in lesion type (1,0,1), rather than type (0,1,1) or (1,1,1) [6, 23]. These results also indicated that it was advisable to analyze the cause of adverse events in clinical data from the standpoint of LDL transport. The results, therefore, indicate that the Medina classification is a classifying method, but does not represent the degree of the disease.

Furthermore, the concentration of LDL along the outer wall of the artery was, on the whole, higher than that along the inner wall. The results provided verification and an explanation for the clinical findings [24, 25], which suggested that the outer wall of the bifurcation lesion SB was more prone to atherosclerosis progression than the inner wall. Based on these, some ingenious devices, which can regulate the flow in order to suppress the accumulation or infiltration of LDL concentrations along the outer wall of the SB, should be invented in the future.

This study also declared that the concentrations of LDL on the surface of the SB significantly increased with the stenosis severity (Figure 6). The models with 75% diameter stenosis were in higher level of LDL than others. These results are consistent with the numerical and clinical findings of others [3, 26, 27]. Koo [26] found that the ratio of flow to the SB dropped with the stenosis severity elevated. In the clinical study by Hahn et al. [3], they reported that severe stenosis was also significantly associated with SB occlusion. Therefore, the necessary intervention should be applied in bifurcation lesions with significant stenosis or occlusion. Moreover, it was found that the lesion type (1,0,1) with a moderate stenosis (50% diameter stenosis) was in a relatively severe condition in the SB due to the higher level of luminal surface LDL concentration. These results indicated that the distribution of LDL could be determined by both the lesion configuration and the stenosis severity. Therefore, it might be unreasonable to carry out the interventional therapy only from the degree of stenosis.

Besides, the water filtration rate represents an essential characteristic of living tissues in the circulatory system. Earlier studies found that the water filtration velocity had significant influence on surface concentration of lipoproteins [28, 29]. It was suggested that elevated water filtration velocity would greatly increase the area of higher surface concentration and the surface concentration of LDL itself [28, 29]. Therefore, new devices or bioprostheses should be designed with a lower water filtration velocity to suppress the accumulation of atherogenic substances in future.

There were still some limitations in this work. The coronary artery bifurcation was simplified as an ideal model with axisymmetric stenosis; however, vessel curvature and bifurcation angle, etc. occurs in different places along artery in the clinical data [30], which could markedly modify the reported results [31]. By employing straight coronary arteries, this work only investigates the influence of the location of lesions on the distribution of LDL. Although an earlier study [32] reported that different LDL concentration initial boundary conditions [33] and outlet boundary conditions would lead to obviously different transport of LDL, there are no proper boundary conditions as in vivo reported in the absence of reference data, specifically, for significantly varied lesions with various regions along the artery. Besides, this work did not employ the effective numerical approaches to simulate turbulence flow in the coronary, even if we are aware that the presence of transitional flow in an arterial stenotic stenosis 50% or more could change the transport of LDL. Due to the computational difficulties, the arterial wall in this work was simplified as a wall-free model, which ignored the mass transfer of LDL in the arterial wall and without considering particle residence time. Therefore, the coronary artery bifurcation-based medical images and more accurate parameters for mass transfer should be carried out in future work.

## 5. Conclusion

We numerically studied the distribution of LDL in coronary bifurcation lesions based on the Medina classification and analyzed the effect of the stenotic side branch (SB) on the luminal surface LDL concentrations. The comparison indicated that the models with the stenotic SB were most likely involved in the higher level of LDL, which might result in more LDL accumulation/infiltration within the SB enhancing the process of atherosclerosis. In particular, these results predicated that the lesion type (1,0,1) was riskier than others lesions. Moreover, the severe stenosis may contribute to aggravating the accumulation of LDL at SB, thus quickening the process of the atherosclerosis. Therefore, both the stenosis severity and geometrical configurations of the coronary branch are absolutely necessary factors for the underestimation of disease severity and corresponding treatment protocols. These results are meaningful for understanding the cause of SB occlusion being recognized as a contributing factor for the etiology of myocardial infarction in clinical data.

## Data Availability

The data used to support the findings of this study are available from the corresponding author upon request.
